# Dysphagia in Lewy body dementia - a clinical observational study of swallowing function by videofluoroscopic examination

**DOI:** 10.1186/1471-2377-13-140

**Published:** 2013-10-07

**Authors:** Elisabet Londos, Oskar Hanxsson, Ingrid Alm Hirsch, Anna Janneskog, Margareta Bülow, Sebastian Palmqvist

**Affiliations:** 1 Clinical Memory Research unit, Dept of Clinical Sciences Malmö, Lund University, Sweden; 2 Diagnostic Center of Imaging and Functional Medicine, Dept of Clinical Sciences Malmö, Lund University, Sweden

**Keywords:** Dementia with Lewy bodies, Parkinson’s disease dementia, Dysphagia, Videofluoroscopy

## Abstract

**Background:**

Dysphagia, which can result in aspiration pneumonia and death, is a well-known problem in patients with dementia and Parkinson’s disease. There are few studies on dysphagia in patients with dementia with Lewy bodies (DLB) and Parkinson’s disease dementia (PDD), especially studies objectively documenting the type of swallowing dysfunction. The aim of this study was therefore to investigate the prevalence, and define the actual swallowing dysfunction according to a videofluoroscopic swallowing examination (VFSE) in patients with DLB and PDD.

**Methods:**

Eighty-two consecutive patients with DLB or PDD in a clinical follow-up program were asked about symptoms of dysphagia. Those experiencing dysphagia were examined with VFSE. Prevalence and type of swallowing dysfunction was recorded.

**Results:**

Twenty-six patients (32%) reported symptoms of dysphagia such as swallowing difficulties or coughing. Twenty-four (92%) of these had a documented swallowing dysfunction on VFSE. Eighty-eight percent suffered from pharyngeal dysfunction.

**Conclusions:**

Almost all DLB or PDD patients with subjective signs of dysphagia had pathologic results on VFSE, the majority of pharyngeal type. This type of dysphagia has not been reported in DLB before. The results have clinical implications and highlight the importance of asking for and examining swallowing function to prevent complications such as aspiration.

## Background

24 million people suffer from dementia worldwide [1]. Lewy body dementia is the second most common form of neurodegenerative dementia after Alzheimer’s disease (AD) [2] and it is a term that can be used to describe dementia with Lewy bodies (DLB) and Parkinson’s disease dementia (PDD) [3]. DLB accounts for 15–20% of dementia cases based on clinical and neuropathological studies [4] and PDD accounts for 3-4% of all dementia cases [5].

In a longitudinal Norwegian study the cumulative prevalence of PDD was 78% after 8 years of PD duration [6]. DLB and PDD are characterized by parkinsonian signs like bradykinesia, postural instability and rigidity well as visual hallucinations, fluctuating attention and alertness, disturbed REM-sleep and neuroleptic sensitivity. Both PDD and DLB patients have impaired visuospatial ability, executive functions and attention [2,3]. The two diagnoses are separated by the time of onset of the cognitive impairment in relation to the motor symptoms. In PDD the motor symptoms precede the cognitive symptoms by at least 12 months, whereas in DLB the dementia precedes or occurs approximately at the same time as the parkinsonism [2].

A common symptom in dementia is difficulty in swallowing – dysphagia. Clinical presentations of dysphagia may be difficulties in chewing or handling specific texture of solids and liquids, and coughing during meals. A normal swallow consists of three phases; oral, pharyngeal, and oesophageal, and is a rapid and well coordinated sequence of almost simultaneous muscle activities [7]. Swallowing and esophageal mobility are involved in complex neuroanatomic networks where among others, the cholinergic nuclei ambiguus and the dorsal motor nucleus of the vagus nerve (DMV) are part. The parasympathetic system is affected in Lewy body disorders with neuronal loss and alpha-synuclein inclusions in the DMV, which is almost consistently affected already in early stages of the disease. This involvement of the DVM results in esophageal dysmotility [8,9].

A swallowing dysfunction can be suspected based on a patients subjective dysphagia symptoms, but can only be verified and characterized via a clinical or/and instrumental swallowing examination. The most often used instrumental techniques are a videofluoroscopic swallowing examination (VFSE) or a fiberendoscopic examination. Although dysphagia can be present in different types of dementia, it is much more common in DLB than in AD [10]. Dysphagia is not only a symptom associated with dementia, but also a well-known symptom in Parkinson’s disease (PD). According to a meta-analysis by Kalf and colleagues, 82% of patients with PD suffer from objective swallowing dysfunction [11] and another study found that it was equally as common in PDD [12].

A severe swallowing dysfunction may often be correlated with the risk of developing aspiration pneumonia [13], and patients suffering from aspiration pneumonia have a higher incidence of swallowing dysfunction verified on VFSE [14]. Aspiration pneumonia is a serious condition with a mortality rate of up to 62% [15]. Fall and colleagues showed that it was the most common cause of death in PD with a doubled prevalence compared with the general population (29% versus 8%) [14].

Based on clinical cases the hypothesis of this study was that DLB patients with dysphagia symptoms have a high prevalence of swallowing dysfunction since spontaneous complaints are infrequent. Therefore, the primary aims of this study were to investigate:

1. How many of the DLB and PDD patients with subjective symptoms of dysphagia had a verified swallowing dysfunction on a VFSE examination, and

2. To describe the type of swallowing dysfunction according to the stage of swallowing.

## Methods

### Design of the study

The study was a clinical observational study.

### Study population

This study was conducted between February 2009 and August 2011 on consecutive patients at the Memory clinic of Skåne University Hospital, in Malmö, Sweden. All patients with a diagnosis of DLB or PDD who visited a senior physician specialized in dementia disorders (E.L.) were included in the study. The patients with DLB fulfilled the criteria of probable DLB according to the 2005 consensus criteria [2]. The PDD patients fulfilled criteria by Emre et al. [3]. The patients were asked the following two questions related to dysphagia: Are you experiencing any kind swallowing difficulties? Do you have coughing problems (daytime or nighttime)? All patients or proxies who confirmed at least one of the questions were examined with VFSE. The regional ethics committee of Lund, Sweden, approved this study.

### Assessment of swallowing dysfunction

The VFSE was performed in collaboration between a radiologist and a speech and language pathologist (MB) according to a routine protocol for VFSE [7]. A digital radiological equipment from Philip mulidiagnost Eleva was used. The patients were seated in an upright position and the examination was performed in lateral projection, with opportunity to visualize the structures from the oral cavity to the oesophagus i. e. at the level from the sixth to the seventh cervical vertebras. Thereby it was possible to visualise the entire swallowing sequence. However, in a VFSE examination focus is on the oral and pharyngeal phases of the swallowing sequence. Every patient had to swallow different textures of solids and liquids for example; fruit pudding, vegetable timbale, meat paté and normal food (chicken and vegetables) cut into small pieces (0,5 × 0,5 cm). All the different test materials consisted of 45 g product mixed with 15 g E-Z-HD barium sulfate, 98% w/w, (Bracco Imaging, Scandinavia AB, Sweden). Given liquids were thickened-, thin and carbonated liquid. The thickened liquid consisted of 100 g fruit puree mixed with 30 g E-Z-HD barium sulfate, 98% w/w. (Bracco Imaging, Scandinavia AB, Sweden). The thin liquid consisted of E-Z-HD barium 40% weight/volume (Bracco Imaging, Scandinavia AB, Sweden). The carbonated liquid consisted of 40% weight/volume E-Z-HD barium (Bracco Imaging, Scandinavia AB, Sweden) mixed with sodium bicarbonate (4 mg Samarin powder), Cederroth International AB, Upplands Väsby, Sweden [7].

All patients were fed the solid test material, and some of the patients were able to drink the liquids themselves. The amounts given to the patients were small and could vary between 3 ml and 10 ml depending on the patient’s ability to participate during the examination and if there was a suspected risk of tracheal penetration.

In order to describe the characteristics of the swallowing sequence in each patient, the oral and pharyngeal phases of the swallowing sequence were analysed.

### Oral phase

(A)  Oral dysfunction was defined as reduced ability to handle the given product orally according to oral motor dysfunction (chewing or tongue dysfunction, for example to transport the food or liquid backwards to pharynx, or poor bolus formation).

### Pharyngeal phase

(B)  Delayed initiation of the pharyngeal swallow (when the swallow of the bolus did not trigger at the level of the faucial arches).

(C)  Pharyngeal retention (residual material in the valleculae and/or pyriform sinuses after swallowing).

(D)  Penetration/aspiration into the airways.

In order to describe the total swallowing dysfunction, the patients received 1 point for each type of swallowing dysfunction verified (A-D) on VFSE. Each patient could get a maximum score of 4 points.

### Statistical analysis

Statistical analysis was performed with use of the Statistical Package for Social Sciences software (version 20.0.0 for MAC, SPSS, Inc., Chicago, Ill). When comparing group differences between continuous variables the Mann–Whitney *U* Test was used (Tables 1 and 2). Chi-square test was used when comparing two dichotomous variables (Tables 1 and 2). A p-value of less than 0.05 was considered significant.

**Table 1:** Demographic data

	**Patients with no symptoms of dysphagia (n = 56)**	**Patients with symptoms of dysphagia (n = 26)**
Age, mean (range)	77 (63–93)	76 (63–91)
Diagnosis (DLB/PDD)	39/17	20/6
Sex (male)	64%	69%
MMSE, median (range)	19 (3–29)	20 (6–29)

There were no significant differences between the groups.

**Table 2:** Prevalence and type of swallowing dysfunction on VFSE

**Variable, n (%)**	**All patients (n = 26)**	**DLB (n = 20)**	**PDD (n = 6)**
Any type of swallowing dysfunction	24 (92%)	18 (90%)	6 (100%)
Oral dysfunction	13 (50%)	10 (50%)	3 (50%)
Pharyngeal dysfunction	23 (88%)	17 (85%)	6 (100%)
- delayed pharyngeal initiation	16 (62%)	12 (60%)	4 (67%)
- pharyngeal retention	13 (50%)	9 (45%)	4 (67%)
- penetration/aspiration	11 (42%)	9 (45%)	2 (33%)
Oral and pharyngeal dysfunction	12 (46%)	9 (45%)	3 (50%)
Total swallowing dysfunction score, median (range)	2p (0–4p)	2p (0–3p)	2p (1–4p)

There were no significant differences between DLB and PDD regarding any variable.

## Results

### Demographics

During the study period 82 patients with DLB or PDD visited the study doctor (E.L.) at the memory clinic and all were included in the study. The median age was 77 years (range 63–93 years). Fifty-nine patients (72%) suffered from DLB and 23 patients (28%) suffered from PDD. The MMSE score ranged from 3 to 29 points with a median of 20 points. The demographics are shown in Table 1.

### Prevalence of dysphagia symptoms

**Figure 1:**
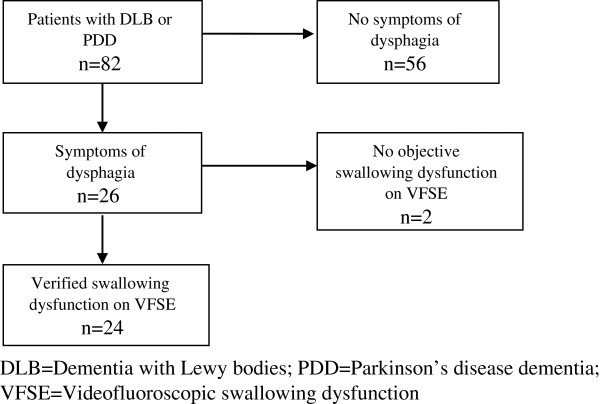
Flowchart of the study population and the prevalence of dysphagia.

Of the 82 patients, 26 (32%) acknowledged dysphagia according to the two questions on swallowing problem and coughing (Figure 1). Twenty of these 26 patients suffered from DLB and 6 from PDD. There were no significant differences in demographic variables between the patient with and without dysphagia.

### Prevalence of objective swallowing dysfunction on VFSE

**Figure 2:**
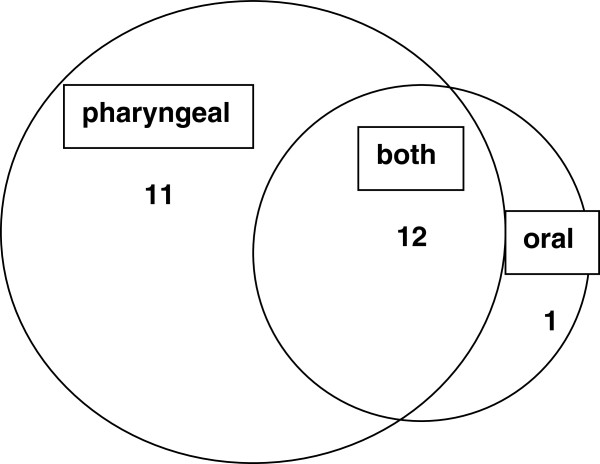
Number of individuals with verified swallowing dysfunction divided according to affected swallowing phase.

Twenty-four of the 26 patients (92%) with subjective signs of dysphagia had a documented swallowing dysfunction on VFSE (Figure 1). Of these 24 patients with a documented swallowing dysfunction, 23 (96%) had a pharyngeal dysfunction and 13 (54%) had an oral dysfunction (Figure 2). Pharyngeal dysfunction was prevalent in 12 of these 13 patients. The occurrence of the different types of swallowing dysfunctions and severity (total swallowing dysfunction score) was almost equal in DLB and PDD patients and no significant differences were found between the groups (Table 2).

### Dysphagia and death due to pneumonia

In February 2013, 12 of the 24 patients with swallowing dysfunction on VFSE had died. The cause of death was specified as pneumonia in 9 of these 12 deceased patients. Eight of the 9 patients with pneumonia had some kind of pharyngeal dysfunction and 5 had oral dysfunction. Among the 56 patients without subjective swallowing dysfunction 13 died, 4 of these in pneumonia, which is significantly fewer compared to those with subjective problems (p = 0.034).

## Discussion

In this study we identified that 92% of patients with Lewy body dementia that acknowledged signs of dysphagia had verified swallowing dysfunction on VFSE. Pharyngeal dysfunction was the most prevalent form (96% of the affected patients). To our knowledge, this study is the first to describe a swallowing dysfunction documented on VFSE in DLB and PDD patients.

The high prevalence of swallowing dysfunction documented on VFSE is consistent with the results of a similar study by Nagaya and colleagues, in which 100% of patients with PD and symptoms of dysphagia had swallowing dysfunction registered on VFSE [16]. Another study has showed that the type of dysfunction in PD is mainly pharyngeal and our study found that this is also the case for DLB and PDD [17]. This differs from the swallowing dysfunction in healthy elderly and AD since these often show an oral dysfunction [18,19]. A reason for this might be that the pharyngeal dysfunction probably is related to Parkinsonism and that the oral dysfunction could be related to dyspraxia (perhaps in combination with more basic features such as reduced sensory input from the bolus). This difference in type of dysfunction also highlights the importance of identifying dysphagia in specifically Lewy body dementia since pharyngeal dysfunction is a higher risk factor for the number one cause of death, aspiration pneumonia. In the present study, we found that 42% of those with subjective signs of dysphagia had penetration/aspiration on VFSE. This is quite alarming when viewed in the light of a study by Yamamoto and colleagues, who found that 83% of patients with aspiration on VFSE developed pneumonia within 2 years compared to 4% in the group without aspiration [20].

Despite the use of quite unspecific screening questions of dysphagia in the present study, all but two patients who confirmed at least one of the questions had objective swallowing dysfunction on VFSE. The high specificity emphasizes the importance of asking about dysphagia when interviewing the patient, and sending all patients with DLB or PDD even with the vaguest symptoms of dysphagia to VFSE primarily to detect those at risk of aspiration. Due to the present study design we cannot present data on the sensitivity of these questions. In a previous meta-analysis on Parkinson’s disease, one third had subjective dysphagia but 4 out of 5 patients had a verified swallowing dysfunction. This suggests that the sensitivity of questions regarding dysphagia is low and that perhaps the majority of patients with Lewy body dementia should be investigated with VFSE.

During follow-up, 12 of the 26 patients with reported dysphagia had died, and at least 9 of them died from pneumonia, probably aspiration pneumonia. This confirms other studies that found a high risk of pneumonia in patients with dysphagia and a high mortality rate in aspiration pneumonia [13-15,21].

A limitation of our study is the relatively small number of studied patients, which makes it possible that this study was underpowered to detect significant differences between groups (Tables 1 and 2). Another limitation is that not all of the patients were investigated with VFSE regardless of the presence of subjective dysphagia. To find the prevalence of objective swallowing dysfunction according to VFSE among patients with DLB or PDD, it would be necessary to examine a larger group of consecutive patients regardless of whether they report symptoms of dysphagia or not. Although the screening questions in our study had a high sensitivity, the use of a validated questionnaire, such as EAT-10 [22], would probably be the most appropriate way to identify dysphagia.

## Conclusions

In conclusion, almost all patients with DLB or PDD in this study with even the vaguest symptoms of dysphagia showed a verified swallowing dysfunction on VFSE, in most cases a pharyngeal dysfunction. This stresses the need of investigating these patients with VFSE and commences adequate therapy and care, especially when considering the fatal consequences of dysphagia and swallowing dysfunction.

## Abbreviations

AD: Alzheimer’s disease; DLB: Dementia with Lewy bodies; PD: Parkinson’s disease; PDD: Parkinson’s disease dementia; VFSE: Videoflouroscopic swallowing examination.

## Competing interests

The authors declare that they have no competing interests.

## Authors’ contributions

EL conceived the idea and recruited the participants. MB performed the VFSE, interpreted and classified the swallowing dysfunction, IAH and AL acquired the data and wrote the first draft, SP supervised and delineated the work. EL, MB, SP and OH analysed and interpreted the data and wrote the final version of the manuscript. All authors read and approved the final manuscript.

## Pre-publication history

The pre-publication history for this paper can be accessed here:

http://www.biomedcentral.com/1471-2377/13/140/prepub
